# Abnormal Presentation and Challenging Diagnosis of Mediastinal Lymphoma: A Case Report

**DOI:** 10.7759/cureus.45668

**Published:** 2023-09-21

**Authors:** Iyad Maqboul, Safaa Abatli, Sara Shbaita, Laith Daraghmeh, Omar Younis, Hanood Abu Rass

**Affiliations:** 1 Department of General Surgery, An-Najah National University Hospital, An-Najah National University, Nablus, PSE; 2 Faculty of Medicine and Health Sciences, An-Najah National University, Nablus, PSE; 3 Department of General Surgery, An-Najah National University Hospital, Nablus, PSE; 4 Department of Pathology, An-Najah National University Hospital, Nablus, PSE

**Keywords:** nodular subtype hodgkin's lymphoma, granular cell tumors, mediastinal mass, classic hodgkin lymphoma, lymphoma

## Abstract

We describe a rare case of classical Hodgkin lymphoma (HL) in a 20-year-old female patient. She presented to our hospital with chest wall swelling after months of post-chest trauma management. The swelling was initially treated as an infected hematoma, and the patient was referred for surgical evacuation. During the surgery, the swelling was found to be a mass that extended to the mediastinum. A biopsy was taken, which later resulted in the diagnosis of a granular cell tumor (GCT). A decision on surgical resection by video-assisted thoracoscopic surgery (VATS) was taken after discussion with the multidisciplinary team of surgery, cardiothoracic surgery, oncology, pathology, and radiology. During the surgery, a frozen section biopsy was taken, which showed no features of lymphoma or granular cell tumors. The surgery was followed by a midline sternotomy to control the bleeding from an accidentally injured major vessel. The bleeding was controlled, and the mass was dissected and sent for histopathological examination. The histopathology showed nodular classical HL features, and the patient was referred for chemotherapy.

In our case, the patient was initially diagnosed with GCT, but with complete resection and an adequate biopsy, the mass was found to be a classical HL. Possible cross-cellularity is questioned, and the possible correlation between the two findings encouraged us to report this case.

## Introduction

Hodgkin lymphoma (HL) is a rare B-cell malignant neoplasm that usually presents with supradiaphragmatic lymphadenopathy, and 33% of the cases are associated with constitutional symptoms, including fever, weight loss, and night sweats. Classical HL is the commonly diagnosed entity of the disease compared to the non-nodular lymphocyte-predominant HL, and it is subcategorized into four definite subgroups: nodular sclerosis, mixed cellularity, lymphocyte depletion, and lymphocyte-rich Hodgkin lymphoma. Nodular sclerosis HL usually affects adolescents and young adults, and it tends to involve the mediastinum and supraclavicular or cervical lymph nodes as a localized disease [1].

The definitive diagnosis of HL requires providing the pathologist with adequate specimens because small biopsy specimens such as fine-needle aspiration and core-needle biopsy may not include sufficient malignant cells [2]. Recently, an overlap of diagnosis between HL and other lymphoma types has been identified, and immunohistochemical studies have been used to discriminate between them [3].

Granular cell tumor (GCT) is a rare soft tissue tumor, commonly considered a benign tumor with less than a 2% malignancy incidence. It is derived from nerve sheath cells called Schwann cells [4]. GCTs can affect all parts of the body, but mediastinal GCTs are extremely rare.

Herein, we present a case of a 20-year-old female patient who presented to our hospital complaining of chest swelling following a trauma. She has been treated for months for trauma complications and wound site infections. The swelling failed to shrink, and the patient underwent a CT-guided biopsy which revealed a diagnosis of GCT. The patient then was referred for surgery to remove the mass, which was found to be a nodular classical lymphoma on histological examination.

The atypical presentation of the HL and the overlap of the diagnosis with the GCT encouraged us to report this case.

## Case presentation

**Figure 1:**
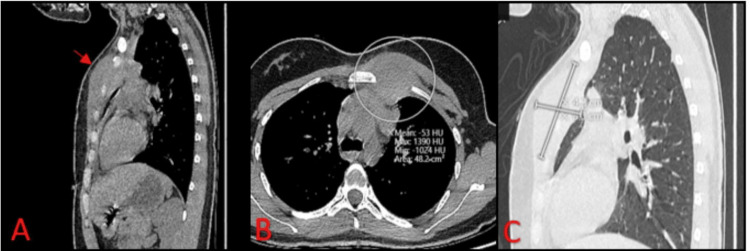
Computed tomography scans of the chest A: the sagittal mediastinal view showed an anterior chest wall mass (red arrow); B: the axial mediastinal view showed an anterior chest wall mass (circle), suggestive of an abscess or liquefied hematoma; C: the sagittal lung view showed the dimensions of the mass to be about 8*4 cm.

A 20-year-old female patient with no past medical and surgical history presented to our hospital with a left-sided chest wall swelling of two months duration after direct trauma to the chest. The patient’s chest swelling was treated at a hospital as a hematoma resulting from the trauma, and it was evacuated. The evacuation failed to reduce the swelling; furthermore, it became tender and erythematous. A chest computed tomographic (CT) scan was done and showed an 8*5*4 cm mass in the anterior chest wall associated with multiple mediastinal lymph nodes with features suggestive of an abscess or a liquefied hematoma(Figure 1). Her white blood cells at that time were 17.9*10^9/L, erythrocyte sedimentation rate was 76 mm/hr, and C-reactive protein was 24.8 mg/L. Chest magnetic resonance imaging (MRI) was also done to rule out other diagnoses, but the findings were suggestive of lymphoma, so the patient was referred to our hospital for chest wall mass excision and biopsy.

The patient underwent mass excision and biopsy, and when the mass was dissected, it yielded dirty fluid filled with cheesy material and necrotic tissue. It was difficult to estimate the size of the mass intraoperatively, but it was about 5*6 cm. After dissection and reaching the pectoralis fascia, the mass was palpated deep into the muscle, for which an incision of the fascia was done, followed by muscle splitting. The mass was located in the retro-muscular area and extended to the anterior mediastinum. Part of the mass wall was biopsied and sent for histopathological examination, which showed connective tissue with dense mixed inflammation and granulation tissue formation with no evidence of malignancy.

**Figure 2:**
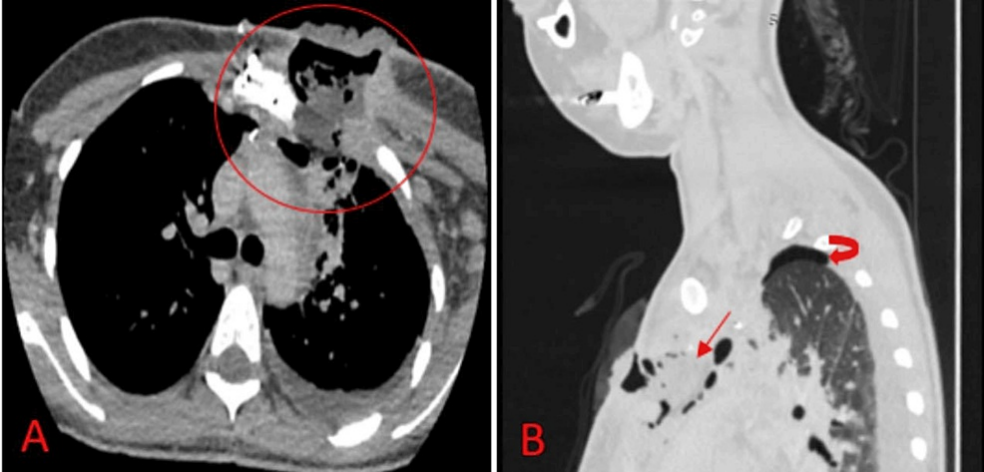
Computed tomography scans of the chest A: the axial mediastinal view showed an infected area around the wound extending deeply into the mediastinum (red circle); B: the sagittal lung view showed the area of suspected infected mass (red arrow), with a pneumomediastinum and a rim of pneumothorax (curved arrow) related to previous surgery.

Following the surgery, the patient developed a wound infection and there was no proper healing. Despite her regular dressings and wound care for about a month, she developed a new collection. A CT scan was done and showed an infected area around the wound extending deeply into the mediastinum, in addition to a suspicious mass located in the retrosternal space (Figure 2). A CT-guided tru-cut biopsy was performed, and it showed a granular cell tumor of the intercostal muscles extending into the mediastinum without any features suggestive of lymphoma. A second opinion was taken, and the diagnosis of granular cell tumor was confirmed by histopathological examination and immunochemistry staining. A meeting of the multidisciplinary team (MDT) of general surgery, cardiothoracic surgery, oncology, and pathology was held to make the decision to perform video-assisted thoracoscopic surgery (VATS) to excise the mass and debride the previously infected wound site.

**Figure 3:**
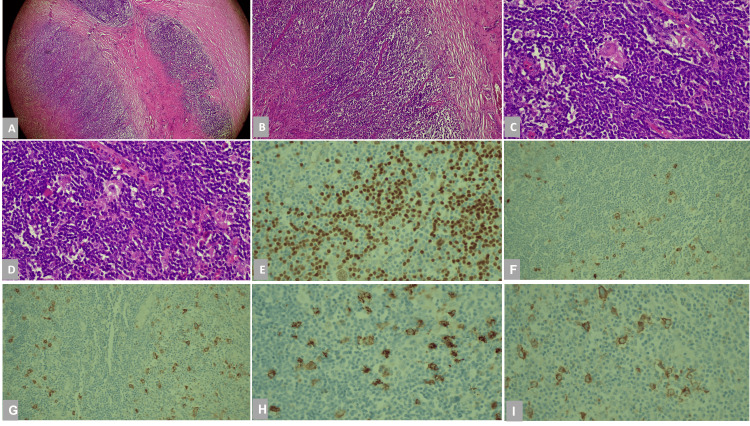
Histopathological examination of the excised mass A. Hematoxylin and eosin (H&E) staining (x40), bands of pink collagenous tissue dividing the field in this lymph node along with Reed-Sternberg cells. B. H&E staining (x10), lacunar cell characteristic for the nodular sclerosis type of Hodgkin lymphoma. It’s a large cell with a surrounding prominent clear space surrounding the nucleus. C. H&E staining (x40). D. H&E staining (x40). E. PAX5 immunostain is expressive of strong immunoreactivity in the small reactive B lymphocyte and stained weakly in the RS cells. F. CD30 immunostain, CD30 positivity in Reed-Sternberg cells, Golgi, and membranous patterns. G. CD30 immunostain. H. CD15 immunostain, positivity in Reed-Sternberg cells, Golgi, and membranous patterns. I. CD30 immunostain.

During the VATS, a frozen section biopsy was taken, and it showed no features suggestive of lymphoma, so the surgery continued. The mass was found to be surrounding the major vessels of the mediastinum; consequently, a small injury to the innominate vein occurred, and the trial of placing a clip to stop the bleeding failed. Thus, the decision to proceed with a midline sternotomy was made to control the bleeding. The bleeding from the innominate vein was controlled successfully. The remaining mass was then shaved from the main vessels and sent for histopathological examination. Debulking of the mass was done, but complete resection was not possible. She had three chest tubes inserted and a vacuum attached to her chest wound. Her vacuum was attached with a pressure of about 80-81 mmHg and was producing a small amount of serous material. It was removed on postoperative day 8. The first chest tube (right chest) was removed on postoperative day 4, the second chest tube (left chest) was removed on postoperative day 6, and the third tube (close to the mass in the anterior mediastinum) was removed on postoperative day 9. The patient was then transferred to the surgical intensive care unit (SICU), where she stayed for three days before being transferred to the surgical ward. The results of histopathological examination of the excised mass were consistent with a Hodgkin lymphoma nodular sclerosis variant (Figure 3). A meeting with the MDT of general surgery and oncology was held, and a plan was made to refer the patient for chemotherapy after recovery from the surgery.

She was referred to the oncology team, and positron emission tomography (PET) was done, which showed stage II lymphoma. She was initiated on a doxorubicin, bleomycin, vinblastine, and dacarbazine chemotherapy protocol about two months after her last surgery.

Her postoperative course was remarkable for a fever with an infected peripherally inserted central catheter (PICC) about three months after her last surgery, for which she had removed it and taken IV antibiotics. This event occurred after her fourth cycle of chemotherapy.

In addition to left upper limb swelling and pain as a result of left upper limb DVT, which was confirmed by a duplex US study of the left upper limb. She was initiated on enoxaparin and then on rivaroxaban. There were no significant complaints except for surgical site pain with the dressing. She was given a course of meropenem and vancomycin for about one week after her last surgery (VATS and sternotomy).

## Discussion

The thoracic mediastinum is the compartment that runs the length of the thoracic cavity between the pleural sacs of the lungs, extending from the thoracic inlet to the superior surface of the diaphragm. The thymus gland, lymph nodes, and connective tissue are among the organs found in the anterior mediastinum. The mediastinum is an essential part of the human anatomy because of its complex structure, which guarantees the appropriate operation of the respiratory and cardiovascular systems.

The mediastinal mass represents a wide entity, referring to a number of benign and malignant diseases that pose a high challenge to most physicians. The mediastinal cavity is divided into anterior, middle, and posterior regions depending on its landmarks. Thymoma, teratoma, thyroid mass, and lymphoma are the most typical causes of an anterior mediastinal mass. Congenital cysts are frequently found in the middle mediastinum while neurogenic tumors are found in the posterior mediastinum [5].

For all types of anterior mediastinal masses, a proper history should be taken, followed by a thorough physical examination. Forty percent (40%) of patients were diagnosed incidentally, with no symptoms. However, symptomatic patients could exhibit either localized symptoms from local invasion, tumor mass effects, or systemic symptoms from the malignancy process itself [6].

If the clinical picture and radiographic findings aren't sufficient, further evaluations can include chest imaging, blood tests, and diagnostic biopsies [7].

Lymphoma is one of the most common etiologies of anterior mediastinal mass, with an approximate incidence of 25% following thymoma, which accounts for 35% [8]. Among all mediastinal lymphomas, only 10% are primary, with Hodgkin lymphoma representing the majority (60%) [5].

Hodgkin lymphomas (HL) are neoplasms arising from the malignant transformation of B-lymphocytic cells. HL is subcategorized into nodular lymphocytic-predominant HL and classical HL. Classical HL has four histological subtypes: nodular sclerosis, mixed-cellularity, lymphocytе-rich, and lymphocytе-depleted [9]. In 2020, the incidence of Hodgkin lymphoma was 98 per 100,000, and mortality was 26 per 100,000. Mortality has decreased since then due to the new therapeutic techniques and improved diagnostic measures, resulting in a marked increase in HL incidence as well. Males were found to be more affected by HL, and a bimodal age distribution has been described as well, with the highest incidence in individuals between the ages of 20 and 40 and a second peak after the age of 55 [10].

Nodular sclerosis classical Hodgkin lymphoma (NS-CHL) represents one of the most common subtypes of Hodgkin lymphoma, comprising approximately 50-70% of mediastinal lymphomas. It is the most common type to involve the anterior mediastinum and has a female predominance. Adenopathy is present in 80% of cases, as seen in this reported case [11].

Patients with mediastinal nodular sclerosis and Hodgkin lymphoma present with various pictures. Patients are usually diagnosed incidentally, as most are asymptomatic. Those with symptoms are mostly present with chest pain, dyspnea, coughing, and fatigue. Superior vena cava syndromе is common as well, and the involvement of adjacent structures may lead to other presentations. Around 30% of patients may exhibit B-symptoms (night sweats, weight loss). Overall symptoms depend on the tumor type, size, site, and stage [12].

Invasion of the chest wall by Hodgkin lymphoma mimicking chest wall tumors has to be kept in mind, as tumor resection has many consequences, including uncompleted tumor resection, post-surgical complications, higher recurrence possibilities, and systemic involvement. Thus, making the right diagnosis is critical to avoiding unnecessary resection. It has been reported in the literature that 15% of first and second-stage cases of mediastinal HL could invade the chest. A study by Witte et al. described four cases of lymphomas presented as chest wall masses. Among these, a 32-year-old male patient presented with a cough and dyspnea. A CT scan showed an anterior mediastinal mass invading the manubrium, and biopsies confirmed the diagnosis of nodular sclerosis HL [13]. This patient presented with nonspecific symptoms and a chest wall mass after being subjected to trauma to the chest. The subsequent persistence of the mass led to investigations that resulted in a nodular sclerosis HL diagnosis.

The diagnosis of HL, like any mediastinal mass, requires clinical evaluation, as mentioned above, followed by laboratory studies. A complete blood count (CBC) could show high white blood cells (WBCs), lymphopenia, normocytic anemia, high lactate dehydrogenase (LDH), and erythrocyte sedimentation rate (ESR) are also seen frequently [7].

Although the most definite diagnosis is achieved by histopathological evaluation, medical imaging plays a crucial role in supporting the CHL diagnosis. A chest X-ray with different views is the first to be done, followed by more sensitive imaging. A computed tomography scan (CT) is the preferred initial modality to determine the exact anatomy, and it plays a role in staging. CT scans in CHL show some specific features, including anterior mediastinal solid masses with irregular borders and surface lobulation but no vascular involvement and associated lymph nodes. In large tumors, invasion of the chest wall or mediastinal structures could be seen. Magnetic resonance imaging (MRI) shows the same findings as the CT scan, with higher inflammatory process detection and fat presence to exclude other differential diagnoses. Positron emission tomography-computed tomography (PET-CT) scans, on the other hand, are used mostly for staging purposes and as recurrence determinants in some challenging cases with post-therapeutic fibrosis [14].

The definitive diagnosis is obtained by an adequate tissue biopsy. Using fine-needle aspiration or even core needle biopsy is usually of low value, as CHL needs good structural evaluation due to the fact that this type of tumor is characterized by a scarcity of malignant cells and abundant inflammatory cells. Nodular sclerosis CHL histopathology shows fibrous tissue surrounding cellular modules and, most times, fibrotic mediastinal lymph nodes [12].

GCTs are rare, commonly benign (two-thirds of cases) neoplasms originating from Schwann cells, affecting adults aged 40-70 years with a female predominance. GCT could occur throughout the body, mostly in the skin or subcutaneous tissue of the head and neck and in the breasts, pulmonary system, and gastrointestinal system [15].

GCT in the mediastinum, on the other hand, is extremely rare, with only a few reported cases in the literature. One Korean study reported a 24-year-old male patient with an incidental mediastinal GCT finding on a chest X-ray [16]. Despite the rarity of chest wall GCT, it has been seen in some of the reported cases. Park et al. described a painless mass at the left lateral chest wall in a 58-year-old female patient after dissection histopathology and immunohistochemical testing revealed benign CGT features [17]. A case has been reported by Gao et al. for a male patient with a right chest mass who presented due to the gradual enlargement of this mass and associated shoulder pain. After investigation, a malignant GCT diagnosis was made [18].

On microscopic examination, these tumors show small nuclear polygonal cells that appear in nests or cords separated by connective tissue with numerous granular cytoplasm. The granules are positive for acid Schiff stain and resistant to diastase. GCT rarely recurs, but in some incompletely resected cases, recurrence and local invasion could occur [19].

The association between CHL and GCT has been reported only in one case of a 13-year-old boy who developed multiple subcutaneous GCT while in HL remission. This could be explained by the fact that HL survivors are at increased risk of developing second malignancies throughout their lives, so the correlation in this case hasn't been proven to be real or coincidental [20]. To our knowledge, no other studies have assessed this correlation.

In our case, the patient had a GCT result for the first biopsies, and with complete resection, the diagnosis of HL was confirmed. Possible cross-cellularity is questioned, and the possible correlation between the two findings urges the need to report this case and complete the patient follow-up in the future.

To summarize our case, a 20-year-old female patient with left-sided chest wall swelling following direct trauma was misdiagnosed as having a hematoma. Later imaging confirmed the presence of a mediastinal mass, further evaluation and biopsy revealed a granular cell tumor, a rare unexpected finding. The patient’s journey continued with surgical intervention and infected wound site debridement until the whole mass was excised and examined. Histopathological examination ultimately confirmed the Hodgkin lymphoma nodular sclerosis variant, and the patient was referred for chemotherapy.

## Conclusions

Understanding the complex structure of the thoracic mediastinum is essential for diagnosing and managing various conditions, including mediastinal masses. Accurate diagnosis of mediastinal masses is crucial, as it influences treatment plans and patient outcomes. While some patients with mediastinal masses may remain asymptomatic, others may present with localized or systemic symptoms, making the comprehensive clinical evaluation essential. Diagnostic approaches for mediastinal masses involve a combination of clinical evaluation, laboratory studies, and medical imaging techniques. However, in some cases like Hodgkin lymphoma, the diagnosis relies on histopathological evaluation through tissue biopsy of adequate samples. Interestingly, the association between Hodgkin lymphoma and granular cell tumors is a rare occurrence, with limited documented cases. This correlation, as observed in our case, underscores the need for further research and follow-up to better understand any potential links between these two conditions.

In summary, the diagnosis and management of mediastinal masses require a multidisciplinary approach, combining clinical expertise, imaging techniques, and histopathological evaluation. Continuous research and reports of unusual cases, such as the one presented here, will contribute to our understanding of these complex conditions and improve patient care in the future.
